# Process analytical approaches for the coil-to-globule transition of poly(*N*-isopropylacrylamide) in a concentrated aqueous suspension

**DOI:** 10.1007/s00216-016-0050-7

**Published:** 2016-11-09

**Authors:** Peter Werner, Marvin Münzberg, Roland Hass, Oliver Reich

**Affiliations:** Physical Chemistry – innoFSPEC, University of Potsdam, Am Mühlenberg 3, 14476 Potsdam-Golm, Germany

**Keywords:** Poly(*N*-isopropylacrylamide), Photon Density Wave spectroscopy, Focused Beam Reflectance Measurement, Turbidity measurement, Particle Vision Microscope measurement, Rate-dependent lower critical solution temperature

## Abstract

The coil-to-globule transition of poly(*N*-isopropylacrylamide) (PNIPAM) microgel particles suspended in water has been investigated in situ as a function of heating and cooling rate with four optical process analytical technologies (PAT), sensitive to structural changes of the polymer. Photon Density Wave (PDW) spectroscopy, Focused Beam Reflectance Measurements (FBRM), turbidity measurements, and Particle Vision Microscope (PVM) measurements are found to be powerful tools for the monitoring of the temperature-dependent transition of such thermo-responsive polymers. These in-line technologies allow for monitoring of either the reduced scattering coefficient and the absorption coefficient, the chord length distribution, the reflected intensities, or the relative backscatter index via in-process imaging, respectively. Varying heating and cooling rates result in rate-dependent lower critical solution temperatures (LCST), with different impact of cooling and heating. Particularly, the data obtained by PDW spectroscopy can be used to estimate the thermodynamic transition temperature of PNIPAM for infinitesimal heating or cooling rates. In addition, an inverse hysteresis and a reversible building of micrometer-sized agglomerates are observed for the PNIPAM transition process.

**Electronic supplementary material:**

The online version of this article (doi:10.1007/s00216-016-0050-7) contains supplementary material, which is available to authorized users.

## Introduction

As a typical member of sensitive or “smart” polymers, poly(*N*-isopropylacrylamide) (PNIPAM) is one of the most frequently investigated polymers within recent decades because of its distinct thermo-sensitive behavior [1–6]. PNIPAM microgel particles exhibit a reversible coil-to-globule transition in aqueous suspensions, induced by a small temperature variation around the polymer’s lower critical solution temperature (LCST). During heating, the swollen polymer microgel particle undergoes a process of changing hydrophilicity at the LCST, inducing a release of water and a compression of the polymer microgel particle. The LCST of PNIPAM is found at approximately 32 °C and thus is close to the human body temperature. Owing to the special thermo-responsive properties of PNIPAM and related materials, its potential for biological and chemical applications has been investigated intensively, for example for drug delivery [7–11], protein separation [12, 13], biosensors [14], or catalyst carriers [15, 16].

The LCST is a fundamental parameter of all temperature-sensitive polymers for the application in chemistry, biology, or material science. For the effective use of PNIPAM-based materials, the adjustment of the LCST to a certain temperature for a specific application is a challenging endeavor. The LCST is affected by many conditions, for example the morphology of the polymer [17], the macromolecular weight and structure as well as environmental factors such as pH, ionic strength [18], and solvent type. It has been shown that the LCST of PNIPAM can be significantly shifted by copolymerization with hydrophilic or hydrophobic monomers such as styrene [19], acrylic acid [20], and others [21].

The LCST can be determined by various analytical methods like optical techniques (e.g., UV-Vis spectroscopy [20–22], dynamic and static light scattering [22–27], infrared spectroscopy [28–31], Raman spectroscopy [32], fluorescence spectroscopy [33]), differential scanning calorimetry [34], or viscometry [35]. However, many of these approaches are usually limited to rather dilute suspensions, exhibiting only single light scattering. In order to access the underlying structural changes within the polymer in its typical environment (i.e., in concentrations typically used for synthesis), further process analytical technologies (PAT), suitable for highly turbid liquids and providing high temporal resolution, are required [36]. Such methods would also be applicable to the synthesis process itself [37]. In this study, the probe-based (fiber)optical technologies Photon Density Wave (PDW) spectroscopy [38–41], Focused Beam Reflectance Measurement (FBRM) [42–45], Particle Vision Microscope (PVM) measurement [46–48], and turbidity measurement [49–51] are applied to study the LCST of aqueous PNIPAM suspensions and additionally its heating and cooling rate dependency.

## Background

### PNIPAM coil-to-globule transition

Aqueous PNIPAM suspensions exhibit a reversible coil-to-globule transition at a specific temperature, the so-called lower critical solution temperature (LCST). At this temperature, the PNIPAM particles undergo a structural change. In literature, different mechanisms of the coil-to-globule transition process of PNIPAM are described [28, 31, 52]. In principle, the mechanism is based on a change of hydrophilicity of the polymer. At temperatures below the LCST, the polymer exhibits an enhanced solubility in water. In this temperature range, each polymer chain is surrounded by water molecules. Near the LCST, the hydrophilicity of the polymer starts to decrease, resulting in a release of water molecules and a compression of the whole polymer network. This causes a change of refractive index during the coil-to-globule transition. During the inverse process (cooling to a temperature below the LCST), the hydrophilicity of the polymer increases again, resulting in an uptake of water molecules and an expansion of the polymer network. Concerning a single PNIPAM particle, the compression and expansion result in a decrease or increase of particle size during the coil-to-globule or globule-to-coil transition process, respectively. It is well known that the coil-to-globule transition exhibits a hysteresis between the heating and cooling period. Depending on the applied investigation method, here the coil-to-globule transition is described to happen mostly at higher temperatures than the globule-to-coil transition process [28].

### Photon Density Wave spectroscopy

PDW spectroscopy is a PAT to quantitatively and independently characterize the absorption and scattering properties of highly turbid liquid suspensions [53, 54]. The absorption coefficient *μ*_a_ and the reduced scattering coefficient *μ*_s_’ are related to the concentration of the light absorbing material and the characteristics of the scatters (e.g., their concentration, size, and refractive index but also their inner particle structure [55]), respectively. In consequence, based on the reduced scattering coefficient, PDW spectroscopy can be used for in-line particle sizing in the size regime of approximately 50 nm to 500 μm [38, 53]. PDW spectroscopy is based on photon transport theory in multiple light scattering systems [56]. It operates in the frequency domain, i.e., the emitted light, creating the PDW inside the multiply light scattering liquid, is intensity modulated with different frequencies. From the dependence of the PDW on the modulation frequency and the distance between emission and detection fiber, the absorption and reduced scattering coefficients are determined independently. In contrast to many other optical technologies, PDW spectroscopy is capable of quantifying the optical properties of the material under investigation at volume fractions of the disperse phase well above 30 %.

### Focused Beam Reflectance Measurement

Focused Beam Reflectance Measurement (FBRM) is a PAT to analyze particle dimensions and particle numbers in liquid suspensions. It is based on the reflectance of a focused laser beam, scanning across the dispersed particles close to the optical probe. Obtained data include the so-called chord length distribution, being sensitive to changes of the particle number and dimension. FBRM is one of the very few in-line probe-based methods for particle characterization in concentrated systems.

### Particle Vision Microscope measurements

PVM is a probe-based in-line microscope, applied to directly visualize suspended structures (e.g., particles, droplets, or cells) within a process. The instrument can be operated in reflection or transflection mode. In addition, a relative backscatter index (*RBI*) can be obtained. The *RBI* is an area-based turbidity signal, deduced from the obtained images.

### Turbidity measurements

Probe-based turbidity measurements exist in various experimental setups, e.g., reflection, transmission (optical density, OD), or transflection geometries are found. Typically, detected light intensities at a wavelength of 860 nm are related to incident light intensities. For quantitative results, especially when comparing to other experimental setups, turbidity measurements require calibration [57].

## Materials and methods

### Chemicals

The monomer *N*-isopropylacrylamide (NIPAM, 97 %, Sigma-Aldrich Chemie GmbH, Taufkirchen, Germany) was recrystallized in *n*-hexane (95 %, Sigma-Aldrich Chemie GmbH, Taufkirchen, Germany) and dried for 24 h before use. The monomer styrene (≥ 99 % Sigma-Aldrich Chemie GmbH, Taufkirchen, Germany) was filtered over aluminum oxide (Carl Roth GmbH + Co. KG, Karlsruhe, Germany) to remove the stabilizer. The crosslinker *N*,*N*-methylenebisacrylamide (BIS, biology grade, AppliChem GmbH, Darmstadt, Germany), the initiator potassium peroxodisulfate (KPS, 99.0 %, Carl Roth GmbH + Co. KG, Karlsruhe, Germany), the buffer disodium phosphate dodecahydrate (≥ 98 %, Carl Roth GmbH + Co. KG, Karlsruhe, Germany), and the emulsifier sodium lauryl sulfate (SDS, 99.5 %, Carl Roth GmbH + Co. KG, Karlsruhe, Germany) were used as received. Ultra-pure water (Milli-Q-Integral system, Merck KGaA, Darmstadt, Germany) was used in all experiments.

### Synthesis of PNIPAM microgel particles

PNIPAM microgel particles were synthesized by free radical precipitation polymerization in aqueous environment according to the formulation in [58–60]. Briefly, 500 mL deionized water was purged with nitrogen under stirring (100 rpm) for 60 min in an automated 1 L lab reactor (OptiMax, Mettler Toldeo GmbH, Gießen, Germany). Both the monomer (NIPAM) (110 mmol) and the crosslinker (0.11 mmol) were added and dissolved. The solution was further purged with nitrogen for 30 min to exclude oxygen, followed by heating-up to 70 °C. An amount of 2.78 mmol of KPS was dissolved in 30 mL deionized water and then injected into the reactor to start the reaction. The polymerization was performed under reflux for 4 h at 70 °C. The polymer suspension was cooled down and stored at room temperature, without further purification. The solid content of the polymer was determined by gravimetry (HR83 Halogen, Mettler Toledo GmbH, Gießen, Germany) to 2.37 % (*w/w*) (conversion 96 %).

### Synthesis of polystyrene particles

Polystyrene (PS) particles were synthesized by free radical emulsion polymerization in aqueous environment. Briefly, 480 mL deionized water was purged with nitrogen under stirring (300 rpm) for 90 min in an automated 1 L lab reactor (OptiMax, Mettler Toldeo GmbH, Gießen, Germany). The emulsifier (4.3 mmol) and the buffer (0.84 mmol) were dissolved in 50 mL deionized water. Both the monomer (908 mmol) and the solution of the emulsifier and the buffer were added into the reactor. The solution was further purged with nitrogen for 60 min to exclude oxygen, followed by heating-up to 55 °C at a heating rate of 1.16 K min^−1^. A total of 3.55 mmol of KPS was dissolved in 4 mL deionized water and then injected into the reactor to start the reaction. The polymerization was performed under reflux for 17 h at 55 °C. The polymer suspension was cooled down and stored at room temperature. The solid content of the polymer was determined by gravimetry (HR83 Halogen, Mettler Toledo GmbH, Gießen, Germany) to 12.54 % (*w/w*) (conversion 74 %, hydrodynamic diameter (*d*_h_) of 110.8 nm (z-average)). For the reference experiments, the PS suspension was diluted with deionized water to 1.34 % (*w/w*).

### Temperature cycling of polymer suspensions

The aqueous PNIPAM suspension, as obtained from the synthesis, was heated and cooled inside the automated lab reactor under stirring (100 rpm) in the range from 25 to 40 °C with ten different rates in the range of 0.1–1 K min^−1^. Temperature equilibration at 25 °C as well as at 40 °C was performed for 20 min. During the experiment, the temperature inside the reactor *ϑ*_r_ and in the reactor jacket *ϑ*_j_ was measured. For the temperature control, the reactor temperature *ϑ*_r_ was used. From the difference between the reactor and jacket temperature *ϑ*_r_-*ϑ*_j_, thermal information is obtained. All temperature cycles were performed for three times in random order. Temperature, PVM measurements, turbidity measurements, PDW spectroscopy, and FBRM were performed simultaneously inside the reactor. Correlation of FBRM, PVM, PDW spectroscopy, and turbidity data with the temperature information of the reactor was provided by self designed software (LabView2011, National Instruments GmbH, München, Germany), assigning a temperature value to the temporal value of an experimental data point (time index provided by the individual instrument software). For PDW spectroscopy, the temporal midpoint of a single measurement was used.

It has to be stressed that the control software of the reactor (iControl 5.2.219 SP1, Mettler Toledo GmbH, Gießen, Germany) is in theory capable of correlating imported data with temperature information. However, the software version 5.2.219 SP1 contains a fundamental bug, resulting in misleading correlation information (see Electronic Supplementary Material (ESM) Fig. S1).

### PDW spectroscopy

The PDW spectrometer is a self-built instrument described in detail elsewhere [61]. Conditions for the PDW experiments were set as follows: emission wavelength of 515 nm, distances between detection fiber and emission fiber of 10–18 mm in 11 steps, intensity modulation frequencies of 10–610 MHz in 41 steps. As the refractive index of PNIPAM could not be determined experimentally, for data analysis [38], the refractive index of PMMA, typical for polymers, was used instead. The refractive index of the suspension is calculated [53] using the refractive indices of pure water (*n*_water_ = 1.3355, [62]) and PMMA (*n*_PMMA_ = 1.4949 [63]) at 515 nm, the solid content of 2.37 % of the polymer, and the densities of water (*ρ*_water_ = 0.997075 [62]) and the polymer (*ρ*_PMMA_ = 1.18 [64]), resulting in a refractive index of the PNIPAM suspension of *n*_suspension_ = 1.3391 at 515 nm. For data analysis of the PS suspension, a refractive index of *n*_suspension_ = 1.3493 was used (*n*_PS_ = 1.6012 [63], solid content of 1.34 %, density *ρ*_PS_ = 1.055 [64]). Due to the unknown exact value of the refractive index of PNIPAM, the obtained optical coefficients are therefore only estimates. However, any changes in the optical coefficients should be related to structural or chemical variations. The temporal resolution was approximately 1 min^−1^. The self-built PDW spectrometer operates well above *μ*_s_’ ≈ 0.05 mm^−1^ [57].

### Focused Beam Reflectance Measurement

The FBRM was performed using a 19 mm probe at 75 Hz (ParticleTrack G400, Mettler Toledo GmbH, Gießen, Germany). Raw data were analyzed applying the “Primary” chord length selection model of the software iC FBRM (Mettler Toledo GmbH, Gießen, Germany). For the particle size analysis, two different chord length fractions were chosen. The first one was chosen for small particles in the range below 50 μm *N*_< 50 μm_ and the second one for larger particles in the range from 50 to 1000 μm *N*_50-1000 μm_. The temporal resolution was approximately 5 min^−1^. The lower detection limit for chord lengths is specified as 0.5 μm by the supplier.

### Particle Vision Microscope measurements

PVM measurements were performed using a 19 mm probe in reflection geometry with a temporal resolution of approximately 30 min^−1^ (V819, Mettler Toledo GmbH, Gießen, Germany). The optical resolution, i.e., differentiation of objects, is specified by the manufacturer as larger than 2 μm. The relative backscatter index *RBI*, describing the relative relation between detected and incident light, was calculated by the software iC PVM (Mettler Toledo GmbH, Gießen, Germany). This parameter can be understood as an area-based turbidity information and is not necessarily limited by the optical resolution given above.

### Turbidity measurements

The turbidity measurements were performed with a fiber-optical turbidity probe (inPro8200, Mettler Toledo GmbH, Gießen, Germany) in combination with a spectrometer (MCS600 with light source CLD 600, detectors MCS 621 and MCS 611, Carl Zeiss AG, Jena Gemany). The relative intensity *I*_R_ at a wavelength of 860 nm in reflection mode was used for turbidity analysis. The temporal resolution was approximately 7 min^−1^.

## Results and discussion

**Fig. 1:**
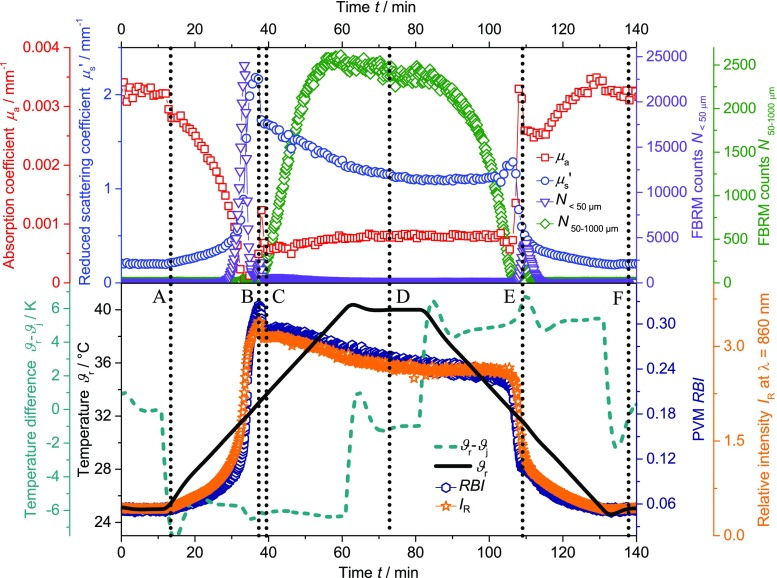
Reduced scattering coefficient *μ*_s_’ (*circle*), absorption coefficient *μ*_a_ (*square*), FBRM counts *N*_< 50 μm_ (*triangle*), FBRM counts *N*_50-1000 μm_ (*diamond*), relative backscatter index *RBI* (*hexagon*), reflected intensity from turbidity *I*_R_ (*star*), temperature *ϑ*_r_ (*solid line*) and temperature difference *ϑ*_r_-*ϑ*_j_ (*dashed line*) as function of time for the PNIPAM suspension (heating and cooling rate of 0.3 K min^−1^). A-F indicate points in time at which PVM pictures in Fig. 4 were taken

Figure 1 shows the reduced scattering coefficient *μ*_s_’, the absorption coefficient *μ*_a_, the FBRM counts *N*_< 50 μm_, the FBRM counts *N*_50-1000 μm_, the relative backscatter index *RBI*, the reflected intensity from turbidity *I*_R_, the temperature *ϑ*_r_, and the temperature difference *ϑ*_r_-*ϑ*_j_ as function of time for one temperature cycle of the PNIPAM suspension with a heating and cooling rate of 0.3 K min^−1^. All applied techniques exhibit signal changes during the heating and cooling cycle of the PNIPAM suspension, especially around the LCST of PNIPAM (for heating at approximately 25–40 min and for cooling at approximately 100–115 min). At the equilibration temperature (25 and 40 °C), most of the signals are constant. This indicates that each of the used techniques is suitable to investigate the coil-to-globule transition of highly concentrated PNIPAM particle suspensions. The temperature difference *ϑ*_r_-*ϑ*_j_ exhibits a short endothermic peak during the heating period (at approximately 35 min) and a short exothermic peak during the cooling period (at approximately 110 min). Therefore, energy consumption and release are limited to a short time period during heating and cooling, respectively. In contrast, these peaks were not observed for the PS suspensions. Hence, it could be supposed that these peaks are connected to the structural changes of PNIPAM. All further peaks of *ϑ*_r_-*ϑ*_j_ result simply from the proportional–integral–derivative (PID) controller during discontinuous temperature changes.

### Coil-to-globule transition of PNIPAM monitored with different techniques

#### PDW spectroscopy

**Fig. 2:**
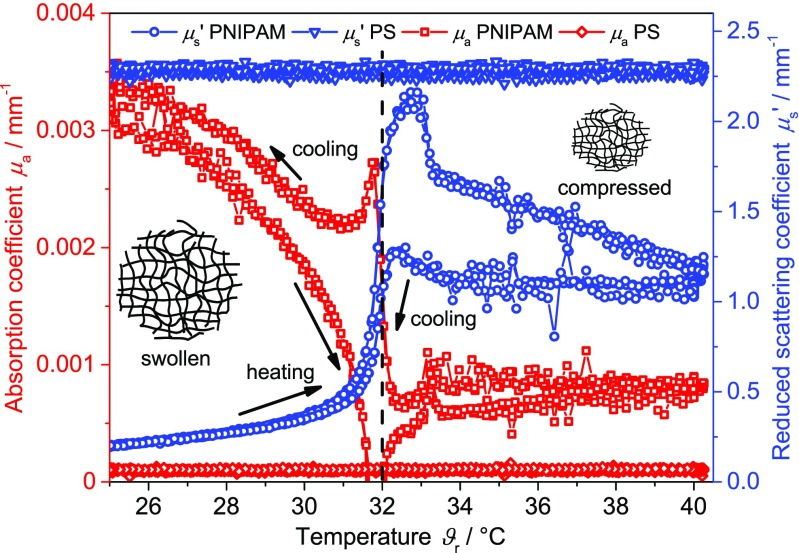
Reduced scattering coefficient *μ*_s_’ and absorption coefficient *μ*_a_ measured by PDW spectroscopy at 515 nm of the PNIPAM suspension (*μ*_s_’ (*circle*), *μ*_a_ (*square*)) and of the PS suspension (*μ*_s_’ (*triangl*e), *μ*_a_ (*diamond*)) as a function of reactor temperature *ϑ*_r_ (heating and cooling rate of 0.2 K min^−1^, 3 temperature cycles). *Dashed line* represents the typical literature value for the LCST of PNIPAM

To obtain specific information about the coil-to-globule transition process of PNIPAM, the signals of the different techniques were plotted against the reactor temperature. Figure 2 displays the absorption and the reduced scattering coefficient of the PNIPAM suspension and the aqueous PS suspension as a function of reactor temperature. Three repeating cycles with a cooling and heating rate of 0.2 K min^−1^ are shown. In case of the PS suspension, no changes of the optical coefficients are observed during the temperature treatment. PS, being a non-thermo-responsive polymer, is expected to undergo no structural changes during the heating or cooling process. Hence, no changes of the optical coefficients are anticipated. Inversely, if changes in the optical coefficients are observed for a thermo-responsive polymer like PNIPAM by PDW spectroscopy, these are most likely due to structural changes of the polymer and are no artifacts of the measurement technology. In case of the PNIPAM suspension, strong changes of *μ*_s_’ and *μ*_a_ are observed. At the beginning of the heating period, in the range from 25 to 31 °C, the reduced scattering coefficient increases from 0.22 mm^−1^ to approximately 0.47 mm^−1^. The reduced scattering coefficient shows a steep increase (to approximately 2.1 mm^−1^) in the temperature range from 31 to 33 °C. Above this temperature range, the reduced scattering coefficient decreases rapidly to approximately 1.7 mm^−1^ within a temperature difference of 0.5 °C, followed by a continuous decrease to approximately 1.2 mm^−1^ at 40 °C. During cooling to a temperature of 33 °C, the reduced scattering coefficient remains constant. Subsequently, after a short increase around the LCST, *μ*_s_’ decreases rapidly to its initial value at 25 °C. In contrast, during heating, a strong decrease in *μ*_a_ is observed already below the LCST. At approximately 32 °C, slightly negative values are determined being unphysical and hence are measurement artifacts. Nonetheless, after the transition of PNIPAM at the LCST, *μ*_a_ quickly equilibrates to approximately 0.6 10^−3^ mm^−1^ and remains nearly constant up to 40 °C. During the cooling period, *μ*_a_ again remains constant until the LCST is reached, followed by a steep increase to approximately 2.7 10^−3^ mm^−1^. Below the LCST, the absorption coefficient increases again to its initial value at 25 °C. Comparing the experimental values for *μ*_a_ at 25 and 40 °C, a change of a factor of approximately 4 is observed. The changes of *μ*_s_’ and *μ*_a_ are believed to be related to the water being removed from the polymer (resulting additionally in a change of refractive index) and to the structural changes of the polymer network when the temperature approaches the LCST. Accordingly, the absorption and the reduced scattering coefficient are potential indicators for the dehydration or hydration status of the PNIPAM particles. As the two optical coefficients are affected differently during heating and cooling, they might be linked to different steps of the coil-to-globule and globule-to-coil transition process. The reproducibility of the two optical coefficients over several heating and cooling cycles indicates that the coil-to-globule and globule-to-coil process of the PNIPAM particles is reversible. The whole transition process of PNIPAM has different impacts on the values of *μ*_s_’ and *μ*_a_. Both the absorption and the reduced scattering coefficients exhibit a hysteresis. To the best of our knowledge, the effect of an inverted hysteresis, as detected by PDW spectroscopy, seems to be no experimental artifact, even though the temporal resolution of the PDW spectrometer limits data quality for the higher heating and cooling rates. The inverted hysteresis has been observed only rarely in literature with other experimental techniques [31]. More often, ordinary hystereses have been described [28, 58, 65–67]. At the moment, the underlying mechanism, i.e., the coil-to-globule transition, being responsible for the inverted hysteresis is still not understood.

#### PVM und turbidity measurements

**Fig. 3:**
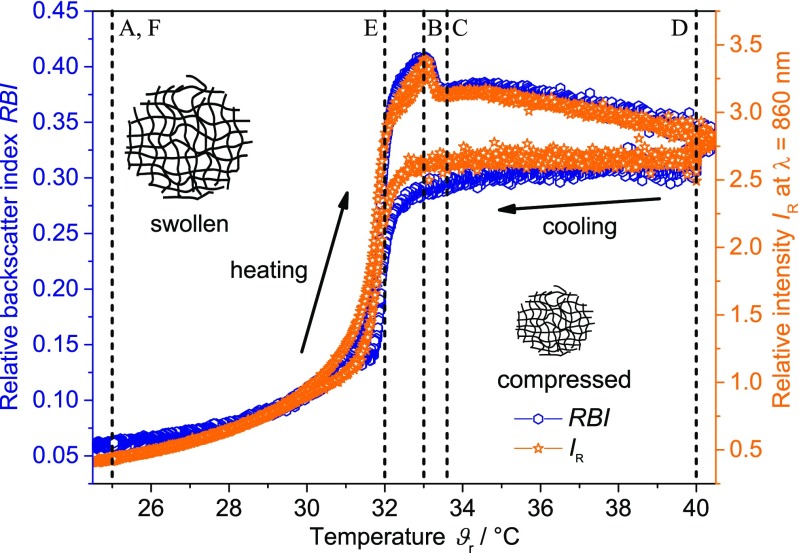
Relative intensity *I*_R_ (*star*) from turbidity measurements and the relative backscatter index *RBI* (*hexagon*) measured by PVM as a function of temperature (heating and cooling rate of 0.3 K min^−1^, 3 temperature cycles for turbidity measurement, 1 temperature cycle for PVM measurements)

In Fig. 3, the relative intensity *I*_R_ of the turbidity measurements and the relative backscatter index *RBI* measured by PVM is displayed as a function of temperature. Three repeating cycles with a cooling and heating rate of 0.3 K min^−1^ for the turbidity measurements and one cycle for PVM measurements (for clearer data visibility) are shown. The relative intensity *I*_R_ of the turbidity probe exhibits a similar trend as the *RBI* of the PVM. In both cases, a continuous increase during heating (25–32 °C), followed by a sharp drop at approximately 33 °C is observed. Subsequently, both signals decrease slightly until a temperature of approximately 40 °C is reached. During the cooling period, both trends exhibit nearly constant values (40–33 °C) until the LCST is reached, followed by a sharp decrease (33–31 °C), and a leveling off to the initial values. Both trends show an inverse hysteresis during the temperature period in accordance to the PDW spectroscopy measurements.

**Fig. 4:**
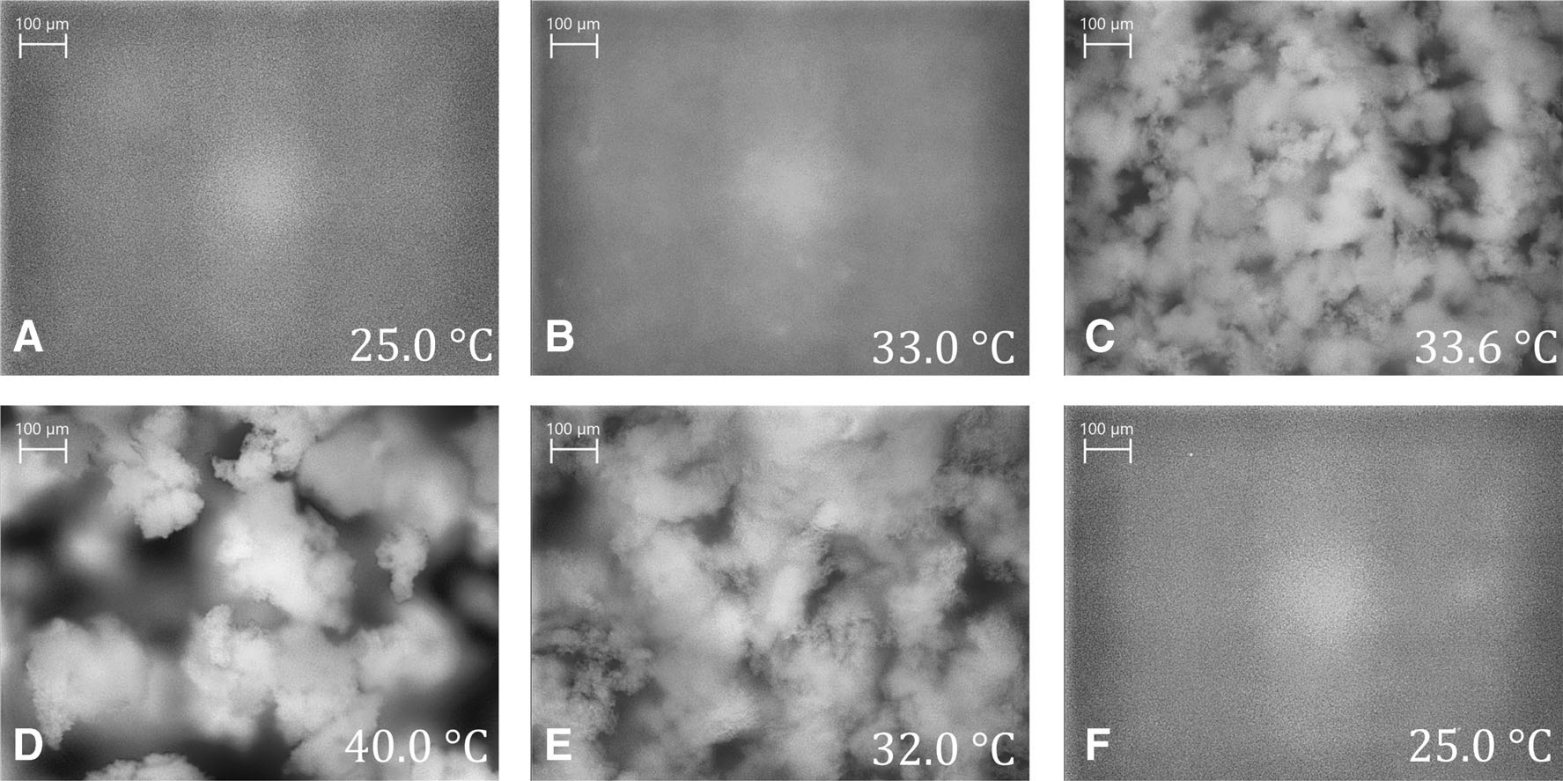
PVM images at specific temperatures (time points A-F, as indicated in Figs. 1, 3 and 5) of the PNIPAM suspension with a rate of 0.3 K min^−1^

For a better understanding of the observed signal changes, corresponding PVM images at significant points in time are shown in Fig. 4 (cf. Fig. 1 for temporal position). At the beginning of the heating period (A, at 25 °C), no structures can be resolved, indicating that the polymer network exhibits no structures in the micrometer regime. The bright spots represent the irradiating light of the PVM probe. Also image B (33 °C, at the maximum value of the *RBI* trend), taken above the LCST during the heating process, shows no structures in the micrometer regime. Surprisingly, only shortly afterwards (C, at 33.6 °C) structures with a floc-like appearance in the size of approximately 100 μm are visible. With increasing temperature, the size of the agglomerates increases to approximately 250 μm (D, at 40 °C). During cooling, the agglomerates disintegrate again (E, at 32 °C), reforming structures at 25 °C (F), which cannot be resolved by the PVM. The detected agglomeration of the PNIPAM particles to these large agglomerates during the heating period therefore seems to be completely reversible. It has been reported that such an agglomeration effect can be induced by adding salt or non-adsorbing polymers [68–70]. In this study, however, the agglomeration occurs without addition of any further substances. Since no purification (e.g., dialysis) of the suspension has been performed, it remains unclear if any residual reactant influences the aggregation step. Before the first and after the last temperature cycle, a STEM image was taken (cf. ESM Fig. S2). In both STEM images, separated non-aggregated particles were visible. No differences were observed, underlying the thesis of a reversible transition process.

Deducing from the PVM images, signal changes of the *RBI* and the relative intensity in the temperature range above 32 °C are probably due to the building of micrometer sized objects. Signal changes below 32 °C are therefore due to processes in the nanometer regime. Here, the sharp increase of the *RBI* and the relative intensity in the range from 30 to 33 °C is believed to display the particle shrinking during the coil-to-globule transition. The release of the water molecules and compression of the polymer result in an increase of the refractive index. This causes stronger scattering and increases the values of the *RBI* and the *I*_R_ as well as simultaneously the reduced scattering coefficient (cf. Fig. 1). Above 33 °C, all three trends exhibit a decrease up to a temperature of 33.6 °C. From PVM images, it can be deduced that the PNIPAM particles start to form larger structures in this temperature range. With increasing temperature, agglomerates are forming with a size of approximately 250 μm. During the cooling period (40–32 °C), the size of the agglomerates decreases again followed by a complete disintegration. The sharp decrease of the three trends (cf. Figs. 2 and 3) is due to the incorporation of the water molecules into the polymer network and hence the swelling of the PNIPAM particles. At 25 °C, no micrometer-sized structures are observed any more.

#### Focused Beam Reflectance Measurements

**Fig. 5:**
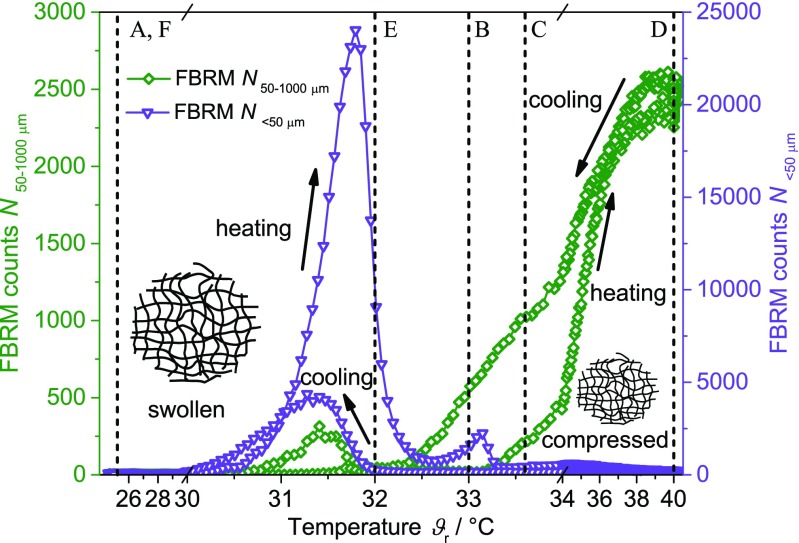
FBRM counts *N*_< 50 μm_ (*triangle*) and *N*_50-1000 μm_ (*diamond*) as a function of temperature (heating and cooling rate 0.3 K min^−1^, 1 temperature cycle)

In Fig. 5, the FBRM counts *N*_< 50 μm_ and *N*_50-1000 μm_ are displayed as a function of temperature. For better data visibility, only one heating and cooling cycle with a rate of 0.3 K min^−1^ is shown. The impact of the coil-to-globule transition on the two FBRM counts is completely different. During the heating period, the number of counts for the small particle fraction (FBRM counts *N*_< 50 μm_) increases temporarily to a maximum at approximately 31.7 °C followed by a decrease back to the initial value at approximately 32.5 °C. Only then the number of counts for the larger particle fraction (FBRM counts *N*_50-1000 μm_) increases continuously, leveling off at higher temperatures. During the cooling period, the FBRM trends exhibit a similar but inverse behavior except that the larger particle fraction shows a small number of counts at approximately 31.5 °C. The trend for the smaller particle fraction exhibits a less pronounced peak during the cooling period.

Regarding the absence of indications for micrometer sized objects in the literature, no changes in FBRM trends were expected. However, the FBRM counts *N*_< 50 μm_ indicate an agglomeration of the particles below the LCST (30.5–32.5 °C). At 33 °C, these structures start to build agglomerates visible in the larger particle fraction *N*_50-1000 μm_ and in the PVM images C–E. FBRM counts increase in the larger particle fraction up to a temperature of 40 °C. During cooling, the agglomerates in the larger particle fraction disintegrate again (40–32 °C). Interestingly, at 32 °C, nearly no counts in the smaller as well as in the larger particle fraction are detected even though the PVM image E displays clearly PNIPAM agglomerates in the micrometer regime. It is unclear why the FBRM cannot detect these structures. Potentially, the scattering of these agglomerates at 32 °C is too weak.

The applied PAT differ by their detection limit with respect to the minimal particle size. The relative intensity, the *RBI*, and the reduced scattering coefficient are probably suitable to detect the coil-to-globule transition in the nanometer regime as well as the agglomeration process in the micrometer regime. With an optical resolution of 2 μm, the PVM images on the contrary allow for a structural understanding of the micrometer-scaled agglomerates. For the FBRM, in-depth investigations need to reveal the meaning of the signal trends around the LCST. However, for all PAT, the experimental findings may represent a superposition of nano particle compression and subsequent agglomeration.

In conclusion, all techniques are suitable to detect the coil-to-globule transition of PNIPAM particles and their agglomeration. Significant changes at specific temperatures are observed, which are probably induced by the change of hydrophilicity of the particles. These changes already occur below and extend above the LCST and represent an inverse hysteresis except for FBRM. From the PVM images, an agglomeration effect of the particles during the transition process is found. However, this process is completely reversible and does not change the size of the individual PNIPAM particles.

#### Investigation of agglomerate building

**Fig. 6:**
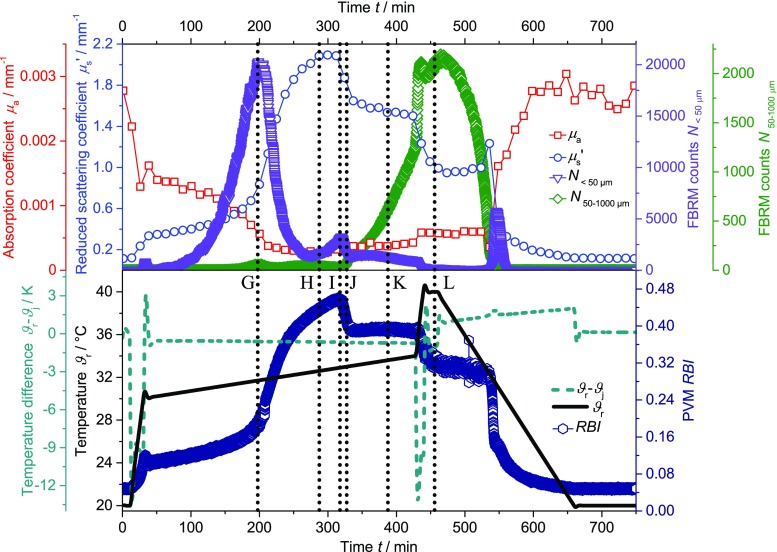
Reduced scattering coefficient *μ*_s_’ (*circle*), absorption coefficient *μ*_a_ (*square*), FBRM counts *N*_< 50 μm_ (*triangle*), FBRM counts *N*_50-1000 μm_ (*diamond*), relative backscatter index *RBI* (*hexagon*), temperature difference *ϑ*_r_-*ϑ*_j_ (*dashed line*) and temperature *ϑ*_r_ (*line*) as function of time for the PNIPAM suspension (heating rate of 0.5 K min^−1^ in the temperature range from 20 to 30 °C, heating rate of 0.01 K min^−1^ in the temperature range from 30 to 34 °C, heating rate of 0.5 K min^−1^ in the range from 34 to 40 °C, cooling rate 0.1 K min^−1^). Points G-L indicate points in time at which PVM images were taken (cf. Fig. 7)

**Fig. 7:**
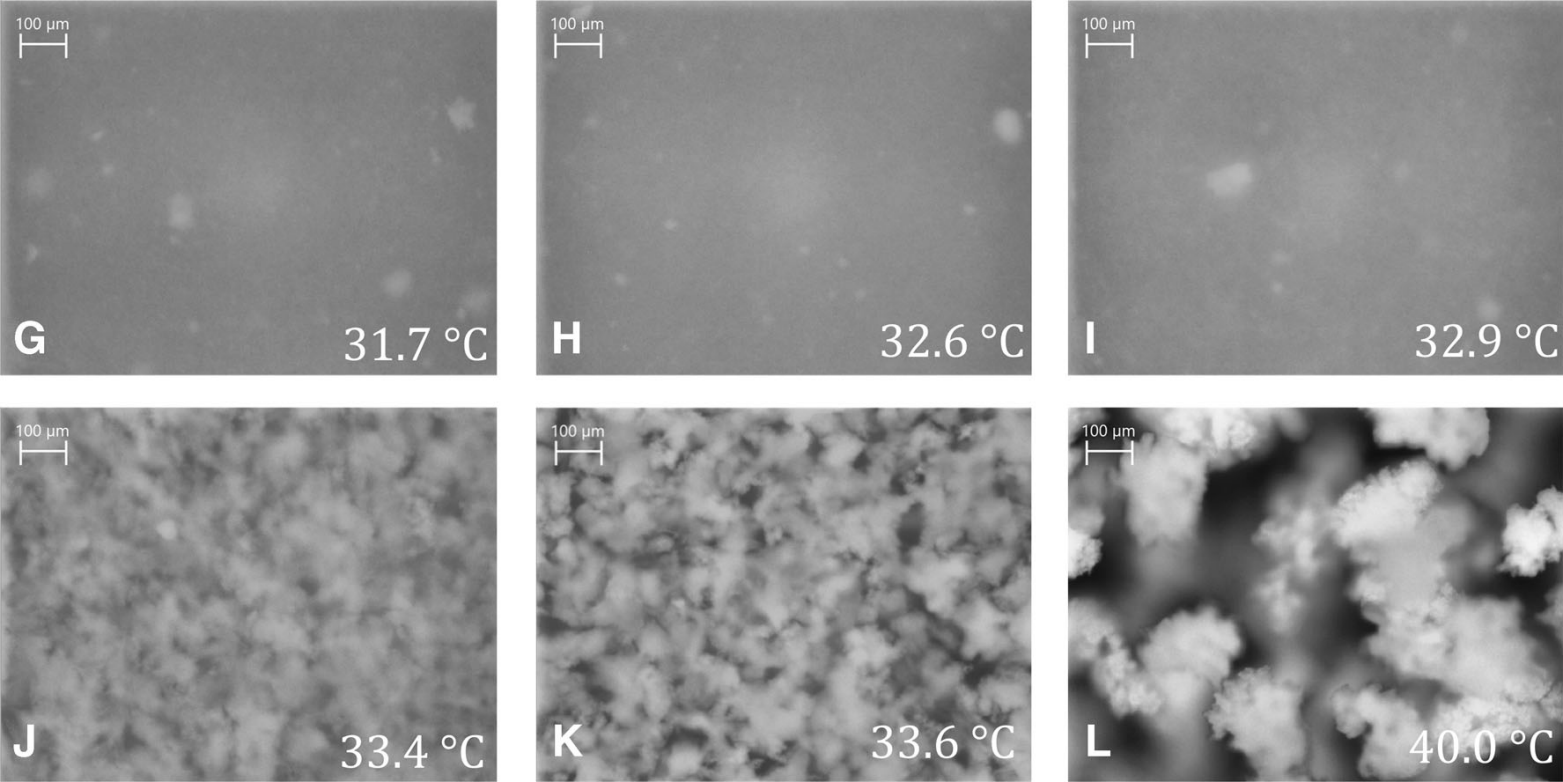
PVM images at specific temperatures (G–L, as indicated in Fig. 6) during heating and cooling (heating rate of 0.5 K min^−1^ in the temperature range from 20 to 30 °C, heating rate of 0.01 K min^−1^ in the temperature range from 30 to 34 °C, heating rate of 0.5 K min^−1^ in the range from 34 to 40 °C, cooling rate 0.1 K min^−1^)

To investigate if the (de-)agglomeration during the heating and cooling cycle is a rate depending effect, the heating rate was reduced to a value of 0.01 K min^−1^. In Fig. 6, the reduced scattering coefficient *μ*_s_’, the absorption coefficient *μ*_a_, the FBRM counts *N*_< 50 μm_, the FBRM counts *N*_50-1000 μm_, the relative backscatter index *RBI*, the temperature difference *ϑ*_r_-*ϑ*_j_, and the temperature *ϑ*_r_ are displayed as function of time. Figure 7 displays the corresponding PVM images at certain temperatures. The heating period starts at 20 °C with a rate of 0.5 K min^−1^. At 30 °C, the system is heated with a slower rate of 0.01 K min^−1^ until the temperature reaches a value of 34 °C, followed by a heating rate of 0.5 K min^−1^ until the system reaches a temperature of 40 °C. The system is held at 40 °C for 20 min followed by a cooling period of 0.1 K min^−1^ until the temperature reaches the initial value of 20 °C.

All trends of the different techniques in Fig. 6 are similar to the trends in Fig. 1. However, for several techniques, a sharp signal change during the change of heating rate from 0.01 to 0.1 K min^−1^ is detected. In detail, the temperature difference *ϑ*_r_-*ϑ*_j_ exhibits an intensive peak resulting from the PID controller of the reactor. Around the LCST, no endothermic peak is detected. The reduced scattering coefficient *μ*_s_’ and the relative backscatter index *RBI* exhibit a signal decrease at the change of the heating rate. Additionally, the number of counts of the FBRM *N*_50-1000 μm_ increases.

During heating, no structures are clearly visible in the PVM images G, H, and I even though *N*_< 50 μm_ and the *RBI* exhibit maxima at approximately 200 min (G, 31.7 °C) and 320 min (I, 32.9 °C), respectively. At approximately 328 min (J, 33.4 °C), the PNIPAM particles start to form larger structures of approximately 20 μm. At the same time, the FBRM counts *N*_< 50 μm_ show a small local maximum. The size of these structures increases with rising temperature (up to image L). Comparing Figs. 4 and 7, the agglomeration process seems to be fast with respect to the change of temperature. The state of agglomeration in Figs. 4B and 7I, 4C and 7K as well as in Figs. 4D and 7L are similar even though the time between the images 4B–D is much shorter (35 min) than between images 7I–L (120 min). It is assumed that the agglomeration starts after the coil-to-globule transition process is nearly finished.

#### Rate-dependent investigation of the coil-to-globule transition

To investigate the rate dependency of the inverse hysteresis, the heating and cooling rates were systematically varied in the range from 0.1 to 1.0 K min^−1^ and randomly repeated three times for each rate. Besides a systematic evaluation of the kinetic behavior, such a variation could allow for the determination of a thermodynamic transition temperature of thermo-responsive suspensions.

#### Rate-dependent PDW spectroscopy

**Fig. 8:**
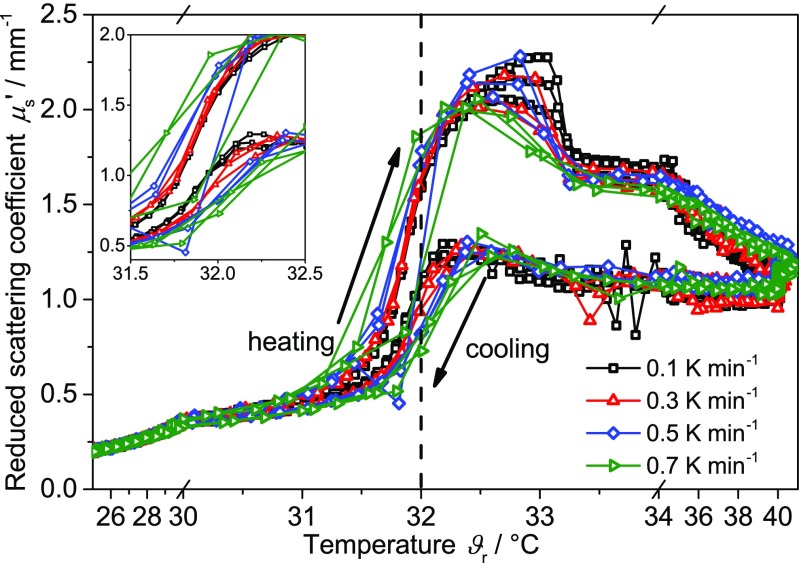
Reduced scattering coefficient *μ*_s_’ of the PNIPAM suspension as a function of temperature at different heating and cooling rates (3 cycles per rate). Inlay: Temperature region between 31 and 32 °C in detail. *Dashed line* represents the typical literature value for the LCST of PNIPAM

In the following, the rate-dependent behavior of changes of PNIPAM will be discussed for each technology. For PDW spectroscopy, this effect is displayed in Fig. 8. The rates were modified between 0.1 and 1.0 K min^−1^ in steps of 0.1 K min^−1^. All cycles were repeated in random order three times (only certain rates are shown for a better data visibility). The increase (heating) and decrease (cooling) of the reduced scattering coefficient is shifted in temperature and represents an inverse hysteresis.

**Fig. 9:**
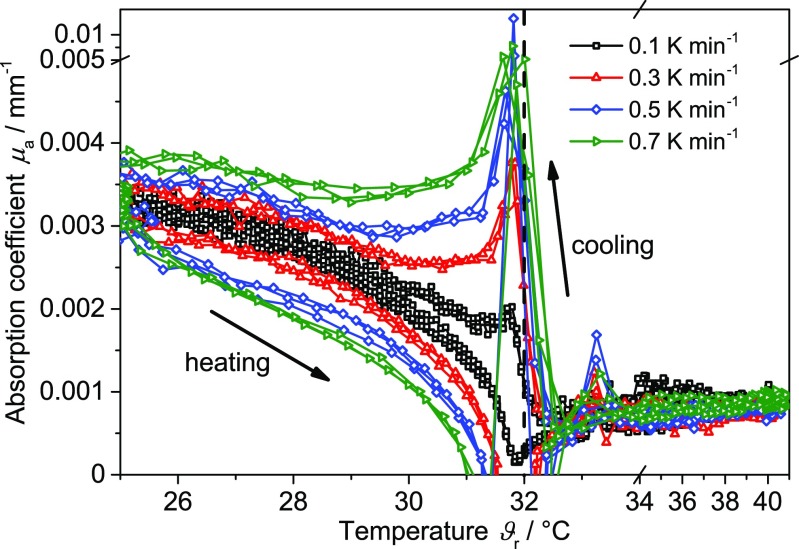
Absorption coefficient *μ*_a_ of the PNIPAM suspension as a function of temperature at different heating and cooling rates (3 cycles per rate). *Dashed line* represents the typical literature value for the LCST of PNIPAM

Figure 9 displays the absorption coefficient as function of temperature, obtained in parallel to the reduced scattering coefficient (cf. Fig. 8). As has been observed also in other studies [33, 58], the transition process of PNIPAM also has a direct influence on the absorption properties. Here, changes of the absorption coefficient are already observed at temperatures of approximately 25 °C during heating as well as during cooling. The maximum (cooling period) increases with higher rate. Hence, the rate dependency of the absorption coefficient suggests a rate controlled hydration or dehydration of the polymer network.

#### Rate-dependent PVM measurements

**Fig. 10:**
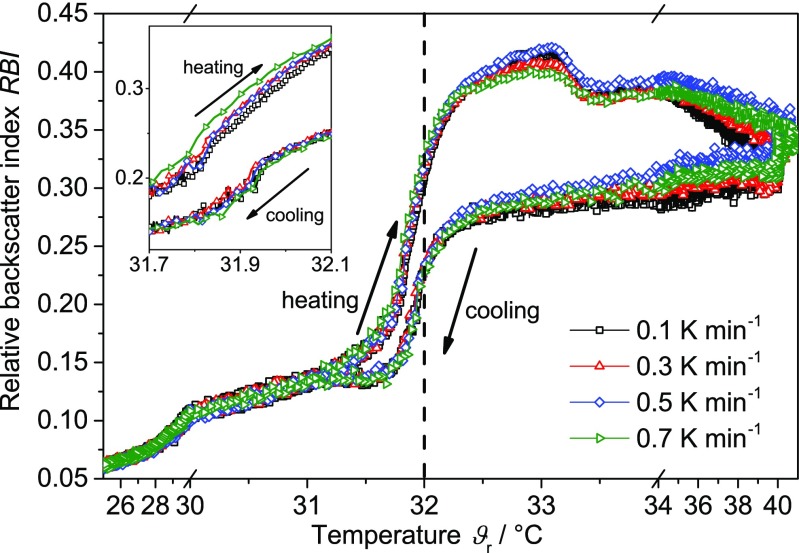
Relative backscatter index *RBI* of the PNIPAM suspension as a function of temperature at different heating and cooling rates (1 cycle per rate). *Dashed line* represents the typical literature value for the LCST of PNIPAM

A rate influence on the hysteresis is also observed for the PVM measurements. Each temperature cycle was repeated randomly three times. Figure 10 displays the *RBI* as a function of temperature with different heating and cooling rates. For better data visibility, the region around the LCST is enlarged. Additionally, only distinct rates and one cycle per rate are displayed. All rates show an increase already below the LCST. The observed hystereses are inverted and slightly broaden with increasing rate.

#### Rate-dependent turbidity measurements

**Fig. 11:**
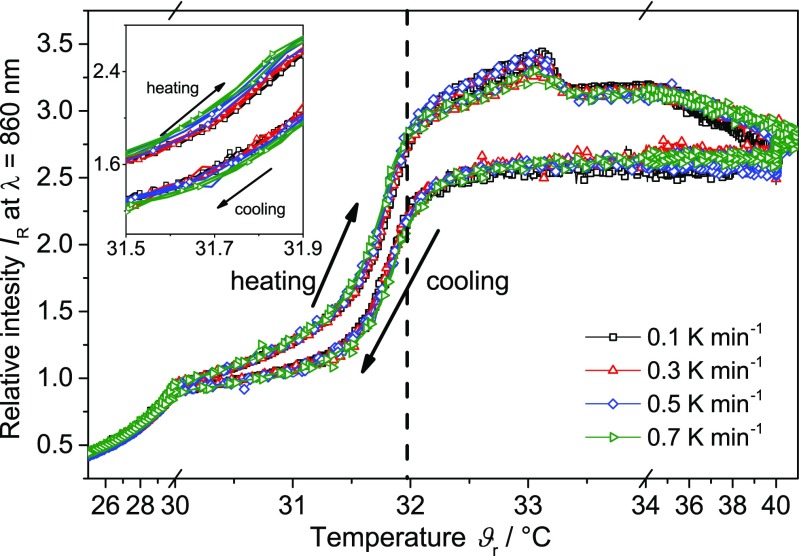
Relative intensity at 860 nm *I*_R_ of the PNIPAM suspension as a function of temperature at different heating and cooling rates (3 cycles per rate). *Dashed line* represents the typical literature value for the LCST of PNIPAM

The rate influence on the hysteresis is also observed for the turbidity measurements of the PNIPAM particles during the heating and cooling period. Figure 11 displays the relative intensity *I*_R_ at 860 nm as a function of temperature at different heating and cooling rates. For a clearer data visibility, only data for certain rates are displayed. The turbidity trends are similar to the *RBI* trends. Both signals exhibit a steep increase (heating) and decrease (cooling) around the LCST. The developed hysteresis exhibits an inverted behavior and a slight broadening with higher rates. The maximum lies at approximately 33 °C for both measurement techniques.

#### Rate dependence of *ϑ*_80_

**Fig. 12:**
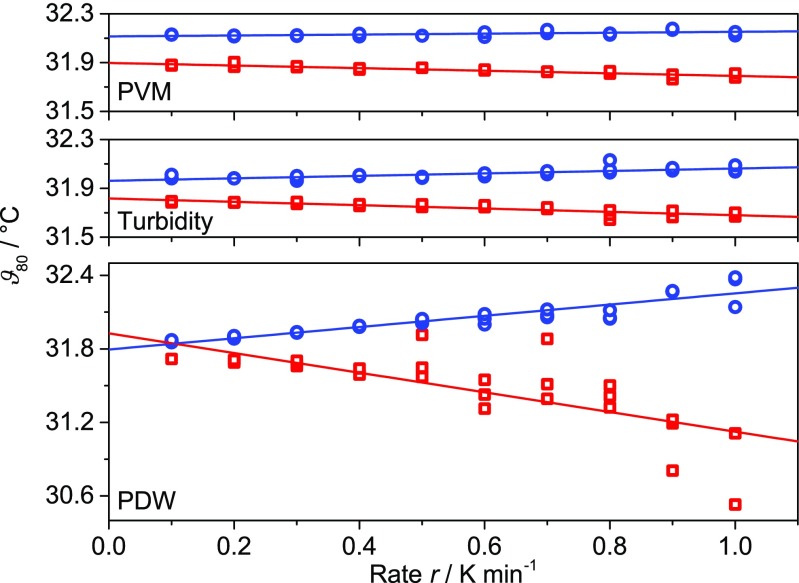
*ϑ*_80_ as a function of heating (*square*) and cooling (*circle*) rate based on the *RBI* value from PVM measurements (*upper graph*), the relative intensity *I*_R_ from the turbidity probe (*middle graph*), and the reduced scattering coefficient *μ*_s_’ from PDW spectroscopy (*lower graph*) for the PNIPAM suspension (3 cycles per rate). Corresponding slopes and intercepts of the linear fits are given in Table 1

The influence of the heating and cooling rate on the hysteresis, as observed by PDW spectroscopy, PVM, and turbidity measurements, shows an interesting effect. For a quantitative comparison, the following definition is used: The minimum and maximum of the signal for the cooling cycle with the lowest rate (0.1 K min^−1^) is determined for each technique. The temperatures *ϑ*_80_ are then defined as those points where the heating and cooling cycles reach a value of 80 % between these minima and maxima (OriginPro 2016G, OriginLab Corporation, Northampton, USA). *ϑ*_80_ has been selected due to a missing analytical fit function describing all complex experimental trends. The chosen value allows for quantitative comparison of the width of the hysteresis around the LCST, across all applied PAT. In Fig. 12, the temperature *ϑ*_80_ is plotted as function of heating and cooling rate. A linear relationship is found for all techniques. The bigger slopes for the heating processes compared to the cooling processes signify a stronger influence of the heating rate on *ϑ*_80_.

Direct comparison of the different techniques indicates that PDW spectroscopy is more sensitive to structural changes (steeper slopes cf. Table 1, intercept for a rate of 0 K min^−1^ cf. Fig. 12) than PVM and turbidity. For a rate close to 0 K min^−1^, the temperature *ϑ*_80_ is linearly extrapolated to (31.9 ± 0.1) °C (maximum error). This value at infinitely small rates could represent the thermodynamic transition temperature between the swollen and the compressed state. For a higher accuracy, rates below 0.1 K min^−1^ would be interesting to investigate.

**Table 1:** Intercepts and slopes with their respective standard errors of the linear fits as shown in Fig. 12

Method	Process	Intercept / °C	Slope / min
PVM	Cooling	32.11 ± 0.01	0.04 ± 0.01
	Heating	31.90 ± 0.01	−0.11 ± 0.01
Turbidity	Cooling	31.96 ± 0.01	0.10 ± 0.02
	Heating	31.82 ± 0.01	−0.14 ± 0.01
PDW	Cooling	31.79 ± 0.02	0.46 ± 0.04
	Heating	31.93 ± 0.08	−0.80 ± 0.14

## Conclusion

Particle Vision Microscope (PVM) measurements, turbidity measurements, Photon Density Wave (PDW) spectroscopy, and Focused Beam Reflectance Measurement (FBRM) have been used as process analytical technologies (PAT) to investigate a concentrated aqueous poly(*N*-isopropylacrylamide) (PNIPAM) suspension during its temperature-induced transition process. As a function of heating and cooling rate, the relative backscatter index *RBI*, the relative intensity at a wavelength of 860 nm *I*_R_, the absorption and the reduced scattering coefficient *μ*_a_ and *μ*_s_’ at 515 nm, respectively, as well as the FBRM counts *N*_< 50 μm_ and *N*_50-1000 μm_ reveal new insights into the transition process of PNIPAM. For all applied techniques, significant changes are found even noticeably below and above the lower critical solution temperature (LCST). In particular, an inverse hysteresis is observed by PVM, turbidity measurements, and PDW spectroscopy, which has been described only rarely in literature. Additionally, a heating and cooling rate dependency has been observed by all PAT. Here, PDW spectroscopy detects the strongest effects. A thermodynamic transition temperature for the thermo-responsive polymer could be estimated to (31.9 ± 0.1) °C by PDW spectroscopy. In conjunction with the change of the absorption coefficient below the LCST, especially PDW spectroscopy may give rise to an increased understanding of the chemical and structural changes during transition processes of thermo-responsive materials in suspension. Furthermore, a fully reversible, clearly temperature-dependent agglomerate formation, which has not been induced by the addition of chemicals, was observed. The agglomerate dimension scales up to several hundreds of micrometers, as e.g. is visualized by the PVM. In addition to conventional sampling-based methods, PVM, turbidity measurement, PDW spectroscopy, as well as FBRM and especially the combination of these different PAT may significantly leverage the development of new thermo-responsive material and the understanding of their transition processes.

## Electronic supplementary material

Below is the link to the electronic supplementary material.

ESM 1 (PDF 359 kb)
