# Supplementary material for: Process analytical approaches for the coil-to-globule transition of poly(N-isopropylacrylamide) in a concentrated aqueous suspension
Source: Anal Bioanal Chem. 2016 Nov 9;409(3):807–19. doi: 10.1007/s00216-016-0050-7 (PMC5233752; doi:10.1007/s00216-016-0050-7)
Supplement: Supplementary file 1 — (PDF 359 kb) [file 216_2016_50_MOESM1_ESM.pdf]

Analytical and Bioanalytical Chemistry

Electronic Supplementary Material

Process analytical approaches for the coil-to-globule transition of poly(*N*-isopropylacrylamide) in a concentrated aqueous suspension

Peter Werner, Marvin Münzberg, Roland Hass, Oliver Reich

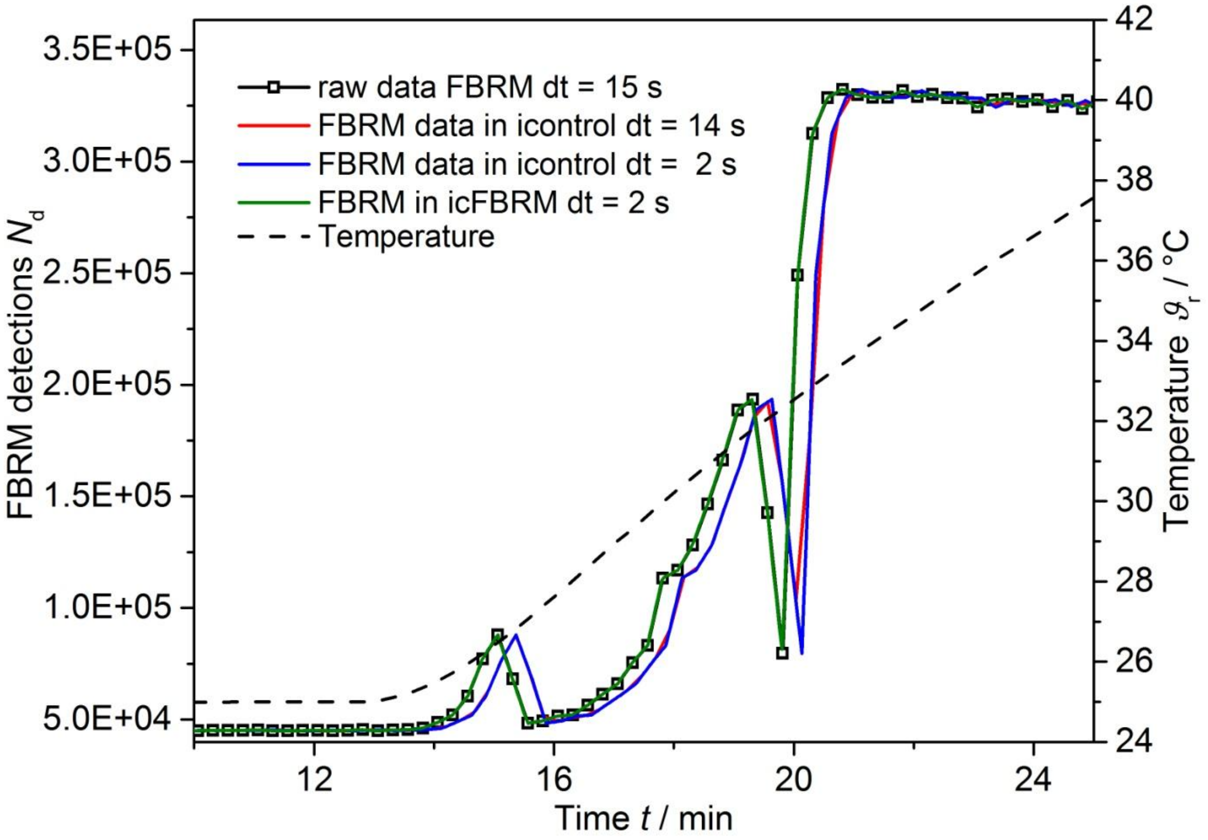

Fig. S1 Bug resulting in misleading correlation information

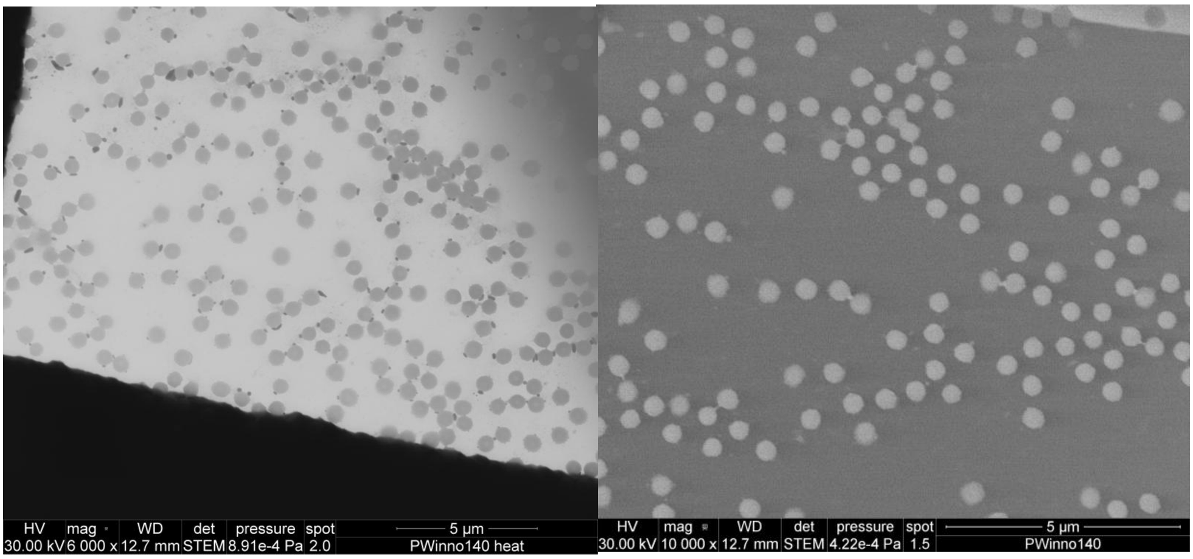

Fig. S2 STEM images of PNIPAM microgel particles before (left) and after (right) the last temperature cycle
